# Regional Differences in Mortality Rates During the COVID-19 Epidemic in Italy

**DOI:** 10.1017/dmp.2020.486

**Published:** 2020-12-22

**Authors:** Danila Azzolina, Giulia Lorenzoni, Luciano Silvestri, Ilaria Prosepe, Paola Berchialla, Dario Gregori

**Affiliations:** 1 Unit of Biostatistics, Epidemiology and Public Health, Department of Cardiac, Thoracic, Vascular Sciences, and Public Health, University of Padova, Italy; 2 Research Support Unit, Department of Translational Medicine, University of Eastern Piedmont, Italy; 3 Department of Clinical and Biological Sciences, University of Turin, Turin, Italy

**Keywords:** COVID-19, ICU, hospitalization, mortality rate, lag effect

## Abstract

****Objective**:**

The coronavirus disease 2019 (COVID-19) outbreak started in Italy on February 20, 2020, and has resulted in many deaths and intensive care unit (ICU) admissions. This study aimed to illustrate the epidemic COVID-19 growth pattern in Italy by considering the regional differences in disease diffusion during the first 3 mo of the epidemic.

****Methods**:**

Official COVID-19 data were obtained from the Italian Civil Protection Department of the Council of Ministers Presidency. The mortality and ICU admission rates per 100,000 inhabitants were calculated at the regional level and summarized by means of a Bayesian multilevel meta-analysis. Data were retrieved until April 21, 2020.

****Results**:**

The highest cumulative mortality rates per 100 000 inhabitants were observed in northern Italy, particularly in Lombardia (85.3; 95% credibility intervals [CI], 75.7-94.7). The difference in the mortality rates between northern and southern Italy increased over time, reaching a difference of 67.72 (95% CI, 66-67) cases on April 2, 2020.

****Conclusions**:**

Northern Italy showed higher and increasing mortality rates during the first 3 mo of the epidemic. The uncontrolled virus circulation preceding the infection spreading in southern Italy had a considerable impact on system burnout. This experience demonstrates that preparedness against the pandemic is of crucial importance to contain its disruptive effects.

The coronavirus disease 2019 (COVID-19) epidemic started in Italy on February 20, 2020, and resulted in a considerable number of deaths (24,648 by April 21). The large number of intensive care unit (ICU) admissions (*n* = 2471 by April 21) and hospitalizations (*n* = 26,605 by April 21) dramatically overloaded the Italian health-care system.^1-3^

The epidemic originated in China and then spread across its borders. On March 11, the World Health Organization (WHO) declared the COVID-19 outbreak to be a pandemic, and Europe became the new epicenter of the disease.^4^

Italy has been severely affected and reported 1 of the most severe outbreaks in Europe in conjunction with Spain. The Italian COVID-19 case fatality rate appeared higher than those observed in other European countries and China. However, this indicator was overestimated in Italy compared with China and other countries because deaths were attributed to COVID-19 disease even when the patients who died had other severe comorbidities.^5^ Moreover, particularly in the early stage of the epidemic, Italy experienced a higher proportion of older patients (>65 y old) with confirmed COVID-19 cases than China; this aspect may also partly explain the differences in case-fatality rates among these countries.^6^

Despite this overestimation, COVID-19 spread heterogeneously throughout the Italian territory, especially during the initial phases of the pandemic, which resulted in many deaths^7,8^ and serious public health problems, particularly in Lombardia, Veneto, and Emilia-Romagna.^9,10^ Some northern areas in particular experienced significantly higher case fatality rates than other regions.^11^ By April 21 in Lombardia, the area most affected by COVID-19, the Civil Protection Department confirmed 12,579 deaths and 851 ICU admissions.^3^

Containment measures of the COVID-19 epidemic, including social distancing, closure of businesses and schools, and a temporary ban on travel, have been implemented since February 23.^9^ These policies were first introduced in the northern regions and subsequently extended to the entire country.^4^

It is, therefore, of striking importance to characterize the Italian COVID-19 diffusion, taking into account the territorial heterogeneity and highlighting the factors that facilitated the uncontrolled virus spreading in the early stages of the epidemic, particularly in the northern regions.^9^

Another challenging issue, considering the differences in COVID-19 diffusion among macroregional areas, is the identification of the most suitable surveillance indicators for the Italian epidemic trend. National and international public health agencies have presented epidemic curves focusing on laboratory-validated COVID-19 cases. However, the epidemic curve representation based on laboratory-validated cases can provide a misleading picture of the disease because this information is affected by different testing criteria and may not be representative of the COVID-19 burden.^12^ In Italy, the COVID-19 testing policy has been heterogeneous over time and in the various regions. During the early stage of the epidemic, tests were performed on suspected patients, ie, hospitalized subjects and individuals who came in contact with positive cases. After this initial phase, only patients with serious symptoms were tested. Recently, tests have also been performed on subjects with no severe signs or symptoms. Moreover, different Italian regions have adopted more or less inclusive testing policies.^13^

Therefore, a meaningful epidemic indicator incorporates a numerator that is minimally influenced by the difference in testing measures and that is adjusted by population size,^2,14^ such as COVID-19 hospitalizations or ICU admissions divided by the resident population or COVID-19 mortality rates per 100,000 inhabitants.^2,14^

In the recent literature, few efforts have been made to characterize and describe Italian COVID-19 epidemic curves during the first 3 mo of the epidemic using opportune surveillance indicators and considering the regional and macroterritorial peculiarities of epidemic diffusion.

This research illustrates the epidemic COVID-19 growth pattern in Italy, taking into account the heterogeneity in virus diffusion and emergency management between Italian regions and territorial macroareas. During the first 3 mo of the epidemic, the disease was characterized by a diffusion pattern in a phase that experienced uncontrolled viral spread in northern Italy with consequent quarantine across the entire country that continued until May 4.^15^

Starting from that date, Italy entered the so-called phase of coexistence with the virus, and restrictions were loosened. Despite a recent increase in infections, Italy is keeping the situation under control compared with other European countries and other nations due to a widespread surveillance and testing system. Between May 1, 2020, and September 9, 2020, Italy registered 76,562 cases, while Spain and France experienced 319,330 and 207,082 infections, respectively.^16^ The worldwide situation remains serious, particularly in the United States, which reported 5,287,884 cases and 128,713 deaths in the same May-September period, followed by India (4,337,078 cases and 72,816 deaths) and Brazil (4,083,911 cases and 121,998 deaths).^16^

This article also aimed to quantify the time series of differences in the epidemic curves among geographical areas during the first wave of the epidemic using indicators that are minimally affected by the testing criteria.

## Methods

### Data Sources

The data source for the numbers of COVID-19 deaths, ICU admissions, and hospitalizations was the Italian Civil Protection Department of the Council of Ministers Presidency^3^; the resident population data were retrieved from the National Italian Statistics Institute.^17^

### Regional Data Description

The mortality and ICU admission rates per 100,000 inhabitants, together with the death/ICU admissions ratio, were obtained at the regional level by defining a design stratified by region and weighting the ratio estimator by the probability of taking the swab test. The many properties of the weighted ratio estimator (no distortion, efficiency, etc.) are specified in the literature.^18^

The regional estimates were summarized with a Bayesian multilevel meta-analysis approach.^19^ Uninformative priors were taken into consideration in the model: (1) A Student’s *t* distribution [*T* ~ (3,1,10)] was used for the intercept parameter, and (2) A Student’s *t* distribution [*T* ~ (3,0,10)] was used for the interregional standard deviation.

The computations were performed by means of the MCMC (Markov Chain Monte Carlo) algorithm with 2000 iterations and 4 chains. The MCMC algorithm convergence was assessed by means of trace plot diagram visual inspection.

The results indicate the following: (1) The time series of observed ICU admissions or mortality per 100,000 inhabitants by region. (2) The forest plots of regional meta-analysis estimates weighted by the probability of taking the swab test, with the 95% credibility interval (CI) and the posterior distributions. This distribution represents the available knowledge about the parameter of interest (ie, the mortality or ICU regional admission rates per 100,000 inhabitants), taking the observed data into account together with prior knowledge (for this research, an uninformative prior T Student’s *t* distribution) of the phenomenon under evaluation.^20^

### Macroarea Data Description

The cumulative COVID-19 mortality rates per 100,000 inhabitants were reported by time (days elapsed since the beginning of the outbreak until April 21) and geographical area (northern Italy and southern Italy). The data referring to the ICU cases and total hospitalizations (per 100,000 inhabitants) were also described by time and geographical area. The data were plotted together with a local polynomial regression smoothing (LOESS) curve^21^ with a degree 2 polynomial approximation and a smoothing parameter of 0.75.

The difference in the epidemic growth pattern between the geographical areas was determined through several indicators: (1) The time series of the difference in COVID-19 event rates per 100 000 inhabitants (mortality, ICU admissions, and hospitalizations) between northern and southern Italy. (2) The time series of the difference in the COVID-19 event rate per day between geographical areas estimated as the daily derivative of the LOESS predicted cases. The 95% CI was also reported. Computations were performed using R 3.6.2.^22^

## Results

### Regional Data Description

**Figure 1.**
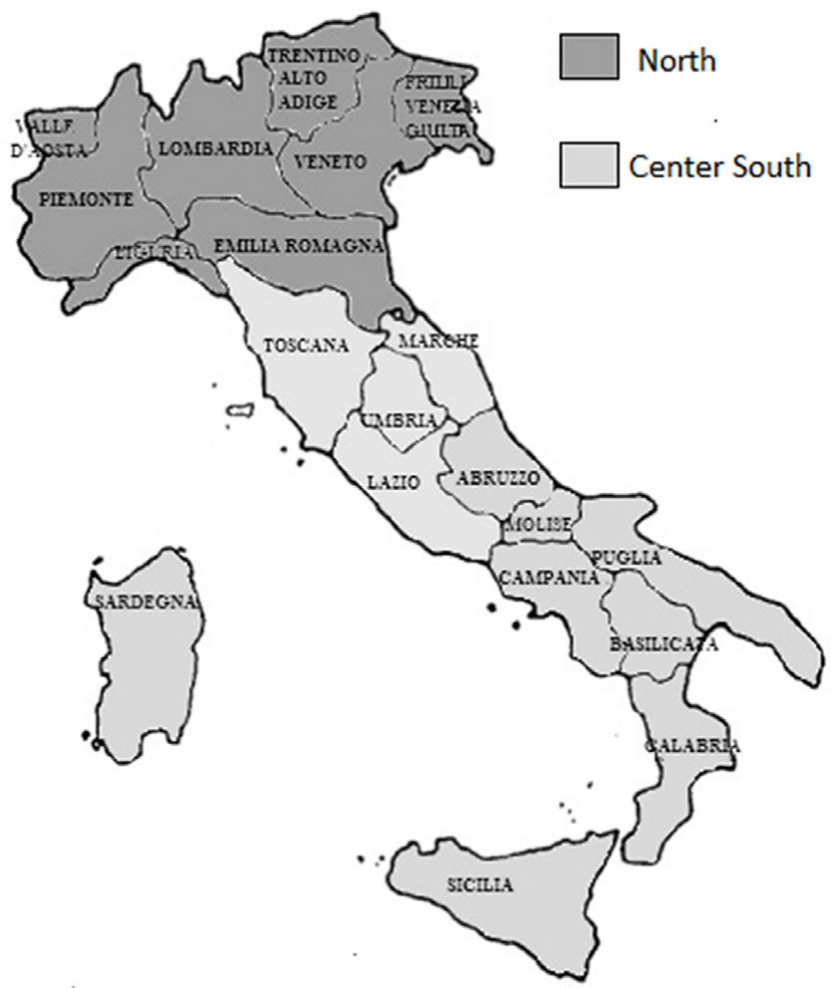
Italian regions map. The northern regions are indicated by dark gray color.

An Italian map with regions and the macro area distinction (North-South) is displayed in Figure 1.

**Figure 2.**
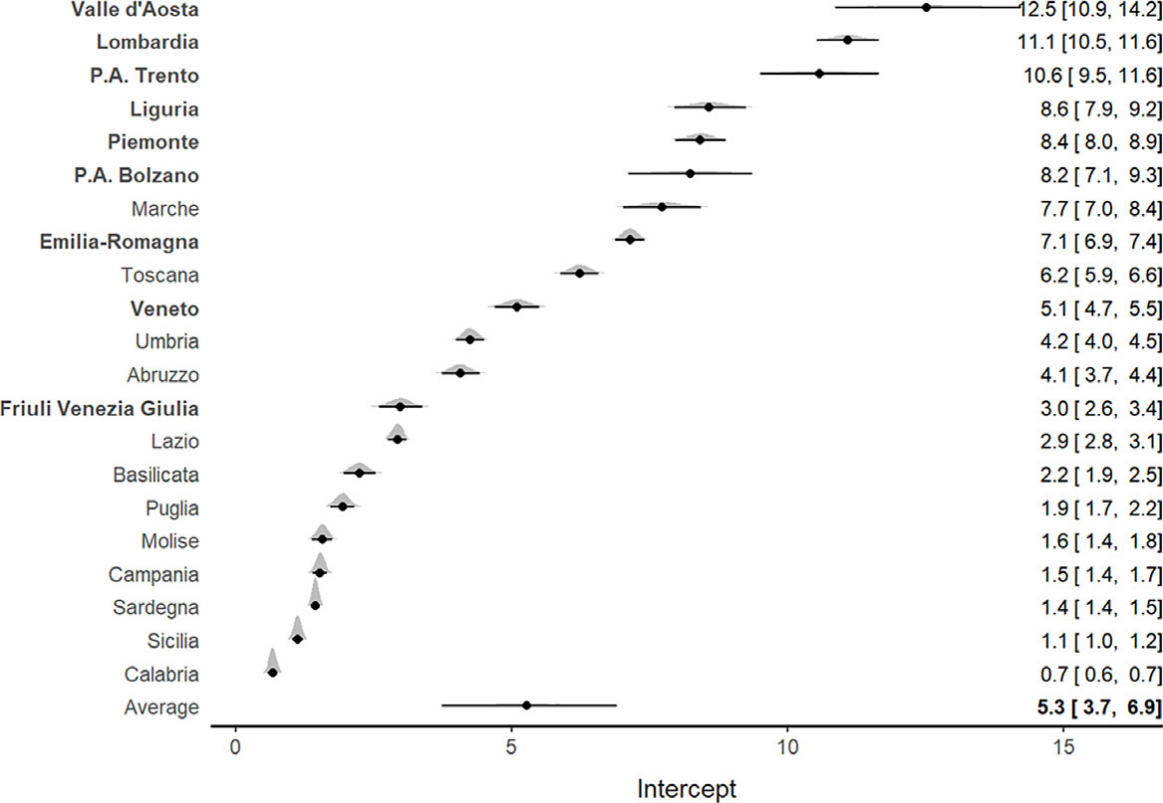
Forest plot for the meta-analytical estimates of ICU admission rates per 100,000 inhabitants weighed by the proportion of swab tests. The posterior distributions together with the 95% CI have also been reported by region. The estimates are reported in decreasing order. The northern regions are indicated by bold text.

The analysis of ICU admission rates per 100,000 inhabitants showed marked heterogeneity at a regional level. The highest admission rates (Figure 2) were observed in the northern regions, particularly in Valle d’Aosta (12.1; 95% CI, 10.5-13.8), Lombardia (10.9; 95% CI, 10.4-11.5), and P.A. Trento (10.3; 95% CI, 9.3-11.4).

A general increasing pattern was detected for the ICU admission rate over time in all Italian regions, with a tendency toward stabilization near the third week of March (Supplementary Figure S1).

**Figure 3.**
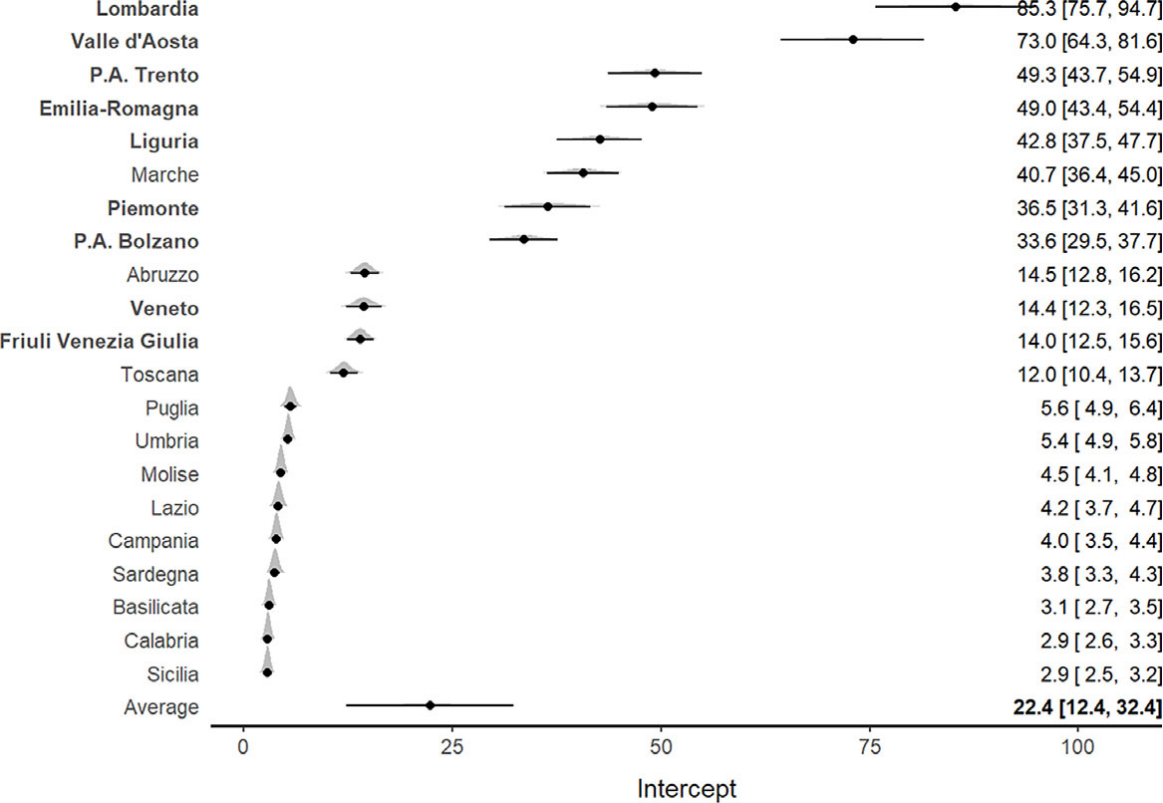
Forest plot for the meta-analytical estimates of mortality rates per 100,000 inhabitants weighed by the proportion of swab tests. The posterior distributions together with the 95% CI have also been reported by region. The estimates are shown in decreasing order. The northern regions are indicated by bold text.

Similar findings were reported for the mortality rate per 100,000 inhabitants. An increasing pattern was observed for all Italian regions, particularly for Lombardia and Valle d’Aosta (Supplementary Figure S2). The meta-analysis (Figure 3) confirmed such results and indicated a higher mortality rate for northern regions, which also showed the highest ICU admission rates, particularly in Lombardia (85.3; 95% CI, 75.7-94.7) and Valle D’Aosta (73.0; 95% CI, 64.3-81.6).

Of interest, Veneto, which was 1 of the 2 Italian regions where the epidemic outbreak started, exhibited both lower ICU admission rates (5; 95% CI, 4.6-5.4) and lower mortality rates (14.4; 95% CI, 12.3-16.5) per 100,000 inhabitants compared with Lombardia (Figures 2 and 3). Southern regions presented the lowest ICU admission and mortality rates.

**Figure 4.**
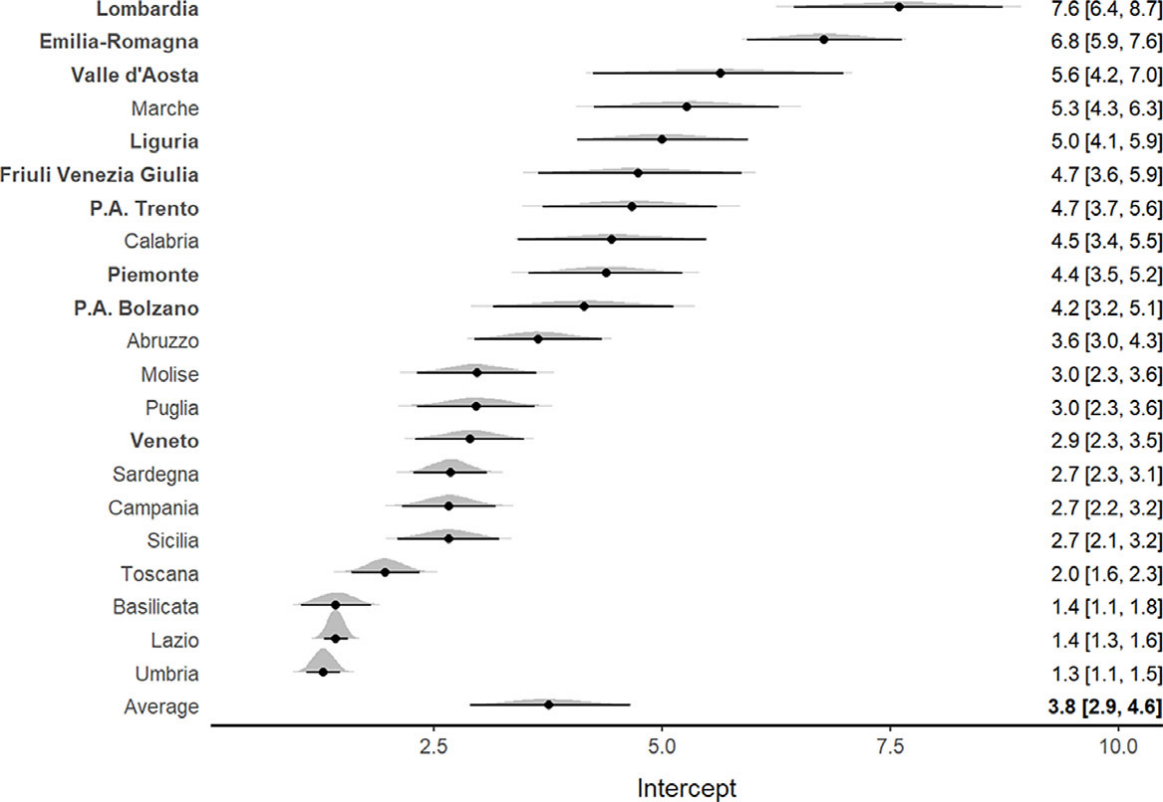
Forest plot for the meta-analytical estimates of the deaths/ICU admissions ratio weighted by the proportion of swab tests. The posterior distributions together with the 95% CI have also been reported by region. The estimates are shown in decreasing order. The northern regions are indicated by bold text.

For the deaths to ICU admission ratio, an increasing tendency was highlighted in all Italian regions (Supplementary Figure S3). Figure 4 shows that the northern regions, characterized by higher mortality rates, had a higher death to ICU admission ratio (particularly Emilia Romagna, Lombardia, and Valle D’Aosta). The trace plot diagrams indicated the absence of patterns among iterations, demonstrating suitable MCMC algorithm convergence (Supplementary Figures S4, S5, S6).

### Macroarea Data Description

The highest mortality rates were observed in northern Italy. An increasing pattern for both geographical areas is shown (Supplementary Table S1, panel 1). The differences in mortality rates per 100,000 inhabitants between macroareas showed an increasing trend, reaching a difference of 66.72 (95% CI, 66.31-67) on April 21 (Supplementary Table S1, panel 2). However, the differences among the daily growth rates peaked in the last days of March and then began decreasing (Supplementary Table S1; panel 3).

The pattern of ICU admissions for both northern and southern Italy increased until the last 10 days of March. A decrease in ICU admissions was observed beginning the first days of April. The highest ICU admission rates were observed in northern Italy, reaching a peak of 11 admissions per 100,000 inhabitants (Supplementary Table S2, panel 1). The difference in ICU admission rates between macroareas increased until the last days of March and then decreased, reaching a difference of 4.02 (95% CI, 3.84, 4.21) admissions per 100,000 inhabitants (Supplementary Table S2, panel 2). The differences in the daily growth rates for ICU admissions revealed a decreasing pattern beginning the second week of March, reaching a difference of −0.18 ICU admissions per day (95% CI = −0.29, −0.06) (Supplementary Table S2, panel 3).

Similarly, the highest hospitalization rates were reported in northern Italy, reaching a peak in the last days of March. In northern Italy, the growth pattern started declining during the first days of April. In southern Italy, a slower decreasing trend was observed (Supplementary Table S3, panel 1). The difference in hospitalizations between geographical areas reached a maximum value near the last days of March and then declined, reaching 54.09 hospitalizations per 100,000 inhabitants (95% CI, 52.45-56) (Supplementary Table S3, panel 2). The daily variation in hospitalization rates between areas peaked during the second week of March and decreased to −1.63 (95% CI, −2.7 - −0.55) hospitalizations per d (Supplementary Table S3, panel 3).

## Discussion

The research findings indicated that there were higher mortality rates during the COVID-19 epidemic in northern Italy compared with the southern regions. This result is certainly related to the disproportionate disease diffusion in some northern areas, which overloaded the health service capacity.^23^

In the literature, some authors have investigated the issues behind the heterogeneity of disease diffusion among Italian regions. Socioeconomic and environmental factors contributed to the differences in the COVID-19 burden in the early stage of the epidemic^5^, together with the timing of the containment policy implementation (Table 1).

**Table 1.** Factors affecting the differences in the COVID-19 burden in the early stage of the epidemic

1. **Socioeconomic factors** a. A historical economic gap between the northern and southern areas has been documented in Italy. A considerable amount of the Southern population still migrates to northern Italy for work and to benefit from higher-quality health-care services in the northern areas (24). b. The Po Valley regions are internally connected for economic reasons by a dense and high-speed network. Most southern cities are excluded from this dense transport network (25). c. Three international airports, frequented by a considerable number of business and tourism visitors, are located in the Lombardy region (26). d. Northern Italy, in particular the Lombardy region, is connected to a dense network of international economic exchanges with other countries, including China (5). 2. **Environmental climatic factors** a. The Po valley, which covers most of the territories of northern Italy, is characterized by elevated concentrations of fine particles due to a considerable number of industries (27). The exposure to this air pollution is associated with chronic lung disease and could constitute a contributing factor to the extreme COVID-19 outbreak in northern Italy (5,28). b. In Northern Italy, indoor activities are preferred, particularly among the elderly, because of the colder climatic conditions during the winter season (26). 3. **Timing of the containment policy implementation** In southern Italy, a limited number of active COVID-19 cases were observed as of March 9, 2020, when the Italian government imposed a national quarantine, restricting the movement of the population except for necessity, work, and health circumstances.

Concerning the socioeconomic aspects, a historical economic gap between the northern and southern areas has been documented in Italy. A considerable amount of the southern population still migrates to northern Italy for work and to benefit from higher-quality health-care services in the northern areas.^24^ The northern regions are, in fact, the economic hub of the country; the Po Valley regions are internally connected for economic reasons by a dense and high-speed network that joins the major cities of Italy from Naples through northern cities, such as Milan and Turin. Most southern cities are excluded from this dense transport network.^25^ The movements between northern regions for work and business may serve as a vehicle for virus transmission in the territory.^5^

Three international airports that are frequented by a considerable number of business and tourism visitors are also located in the Lombardy region,^26^ which is connected to a dense network of international economic exchanges with other countries, including China, where the virus first appeared.^5^

Environmental factors may also play a role. The Po valley, which covers most of the territories of northern Italy, is characterized by elevated concentrations of fine particles due to a considerable number of industries and a particular climatic environment.^27^ Exposure to this air pollution has been associated with chronic lung disease and a decreased lifespan and could constitute a contributing factor to the extreme COVID-19 outbreak in northern Italy.^5,28^

The climate may also contribute to the heterogeneous spread of the virus. Indoor activities are preferred in northern Italy, particularly among the elderly, because of the colder climatic conditions during the winter season.^26^ Moreover, the epidemic started to spread in the last days of January, facilitating social activities in an indoor setting, which led to easier viral and bacterial transmission.^5^

This work shows an increasing differential in mortality rates between the Italian macro areas. The containment measures seemed to flatten the mortality rate curve only in central-southern Italy.^29^ This effect is likely related to the time that the national lockdown measures were implemented. In southern Italy, a limited number of active COVID-19 cases were observed as of March 9, 2020, when the Italian government imposed a national quarantine, restricting the movement of the population except for necessity, work, and health circumstances.

Timing proved to be a crucial factor in influencing the impact of containment measures.^29^ The uncontrolled virus circulation in northern Italy, which preceded the spread of the infection in southern Italy, and the consequent “surprise effect” had a considerable impact on health-care system burnout. One of the lessons learned from the Italian experience in the early stages of the epidemic is that preparedness against infectious outbreaks is of crucial importance. A rapid and efficient contact tracing procedure and a high rate of testing are important to help mitigate and confine the infections; these measures rapidly inform the infected subjects, minimizing the possibility of transmitting the infection.^5^ This issue was also confirmed by the experience of the Veneto region, which, despite being 1 of the first regions affected by the epidemic, quickly contained the effects of the pandemic. The policy of broad-spectrum testing and rapid contact tracing has limited the number of ICU admissions compared with the other northern regions.^2,30^

This study highlights an increase in COVID-19 ICU admissions for both northern and southern Italy until the last 10 days of March, followed by a decrease at the beginning of April. Northern Italy had the highest admission rates. This could be explained by the asynchronicity of the outbreaks in the different Italian regions. Indeed, the virus started spreading in the southern regions almost 2 wk after the northern regions, when severe containment measures had already been implemented on a national level.

The ICU admission rate indicator is less affected by the lag effect than the mortality rate indicator. The literature shows that the median time from symptom onset to ICU admission is 10.5 d (approximately 7 d less than the duration from symptom onset to death).^31^ This shorter lag time likely indicates evidence of the first effects of the containment measure in both northern and southern Italy because the ICU admissions are the result of a contagion shifted back by a shorter time in comparison with mortality.

Northern Italy showed higher hospitalization rates, peaking in the last days of March. The lag effects were more contained for hospitalizations than for ICU admissions; the median time from symptom onset to the first hospital admission was in fact 7.0 d.^32,33^

### Study Limitations

COVID-19 mortality monitoring should also consider the age-specific structure of the population under study. The infection-related death toll is higher for the elderly; therefore, considering the age structure of the population may help explain the differences in fatality rates across different countries or geographical areas. The COVID-19 pandemic appears to behave differently for populations with similar sizes but different age structures, showing a dramatically higher mortality rate in countries with older populations.^34^ Italy has 1 of the world’s oldest populations, with 23.3% of subjects aged over 65 years compared with only 12% in China.^35^

In Italy, excluding some territorial peculiarities, the regional age-specific structures are similar.^36^ This aspect supports summarizing the growth pattern for COVID-19 deaths considering the overall mortality rates, without performing an age-specific standardization.

## Conclusions

Higher and increasing mortality rates during the COVID-19 epidemic were observed for northern Italy, concurrent with a growing differential between the 2 Italian macro areas. A decrease in COVID-19 ICU admissions and hospitalizations for both northern and southern regions was observed, starting from the first days of April, with marked heterogeneity at the regional level.

The shorter lag effect provided by ICU admissions and hospitalizations (compared with death) helps to better understand the possible effects of the containment measures according to geographical areas.

## Data Availability

The datasets used and/or analyzed during the current study are available from the corresponding author upon reasonable request.

## Supplementary material

For supplementary material accompanying this paper visit https://doi.org/10.1017/dmp.2020.486. click here to view supplementary material
