# Supplementary material for: Regional Differences in Mortality Rates During the COVID-19 Epidemic in Italy
Source: Disaster Med Public Health Prep. 2020 Dec 22:1–7. doi: 10.1017/dmp.2020.486 (PMC7985634; doi:10.1017/dmp.2020.486)
Supplement: Supplementary file 1 [file S1935789320004863sup001.docx]

***Supplementary Material***

Table S1 Differential in mortality rates per 100 000 inhabitants among Northern and Southern Italy.

| 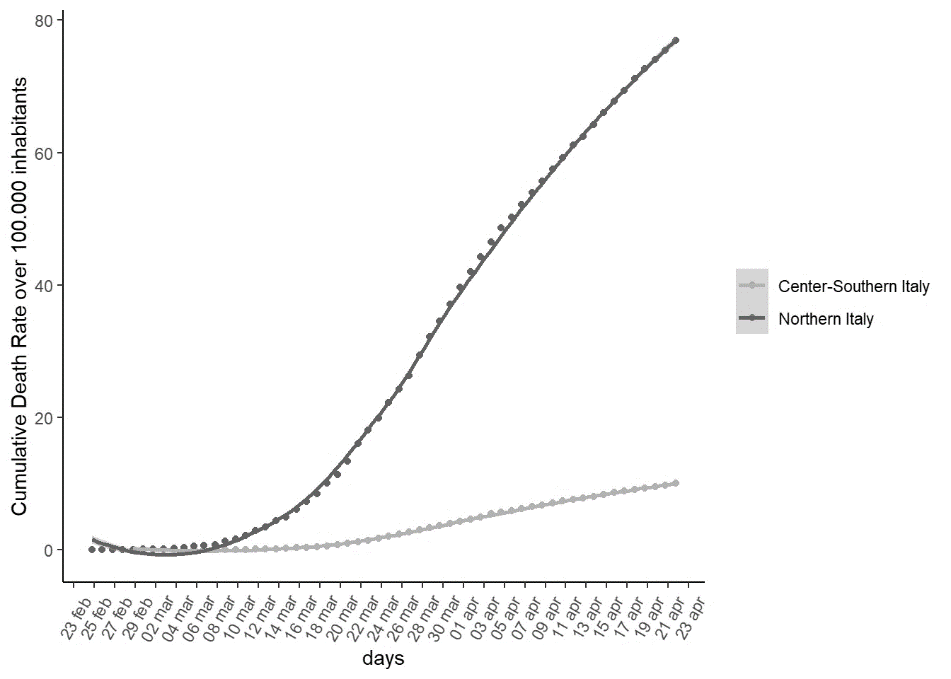  **Panel 1** Cumulative mortality rate over 100 000 inhabitants according to time in days and geographical area. A Local polynomial regressive smoothing spline has been also reported | |
| --- | --- |
| 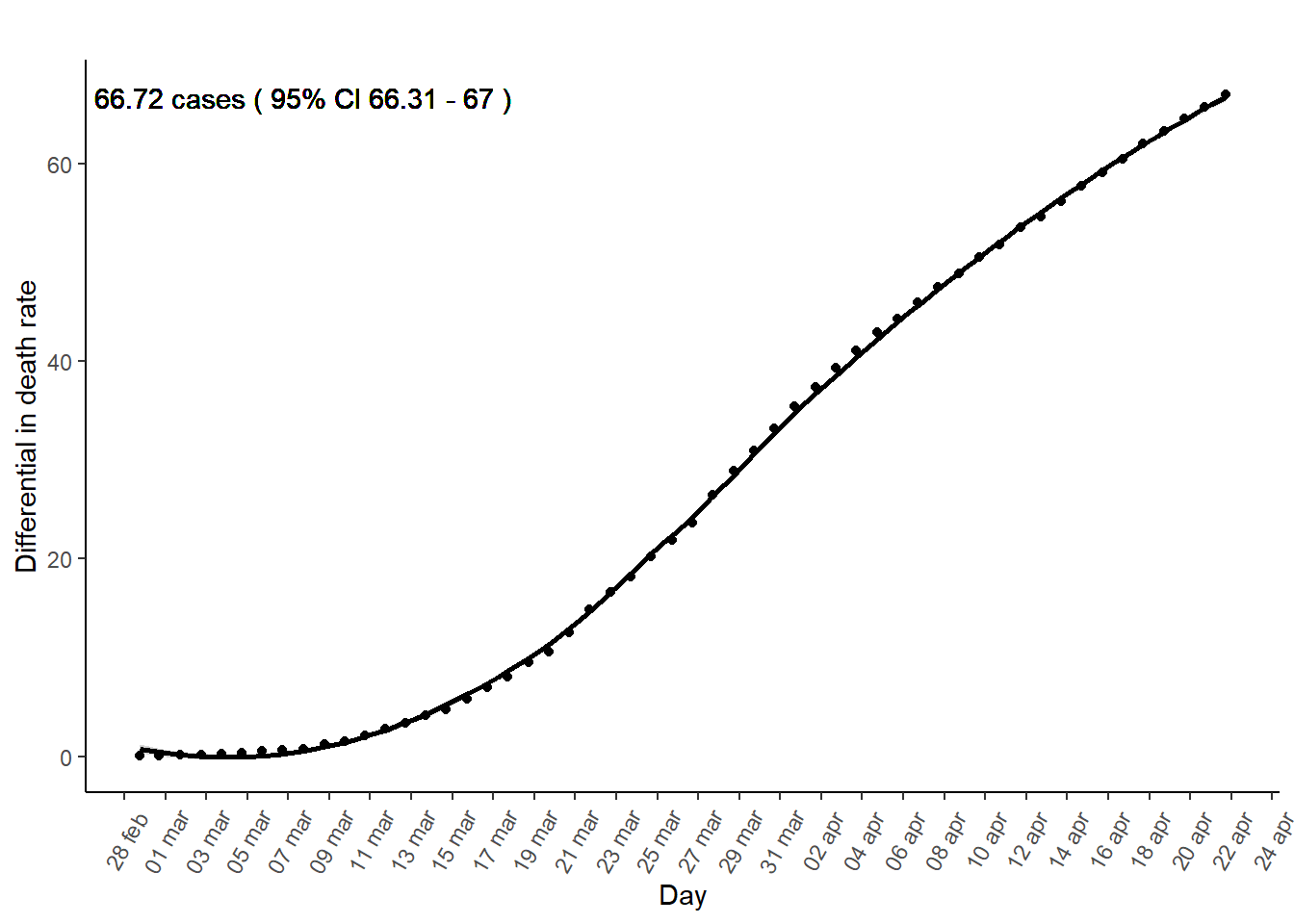  **Panel 2** Time series of the differential in mortality rate between Northern and Southern Italy | 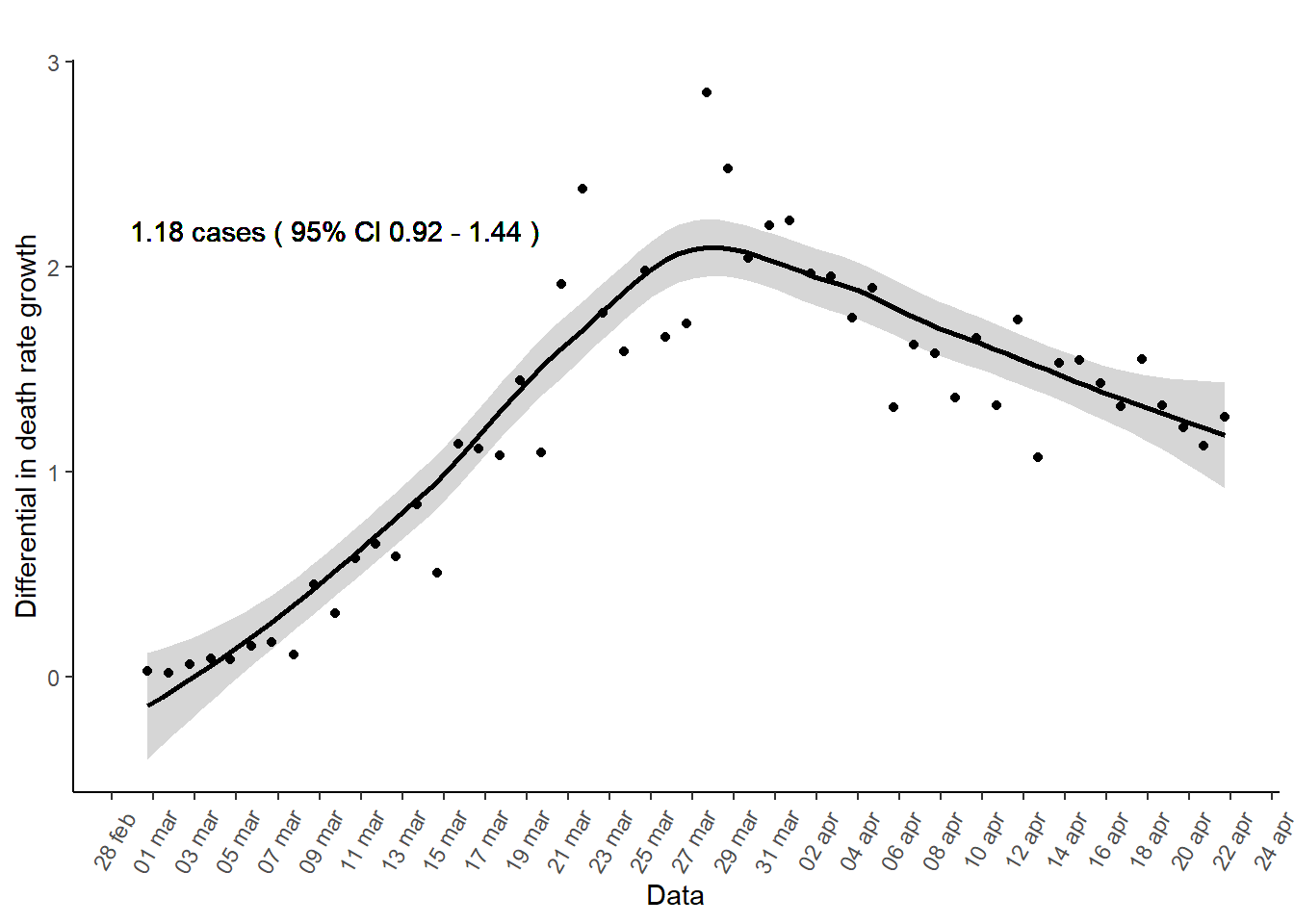  **Panel 3** Time series of the differential in mortality rate daily growth between Northern and Southern Italy. |

Table S2 Differential in ICU entrances rates among Northern and Southern Italy.

| 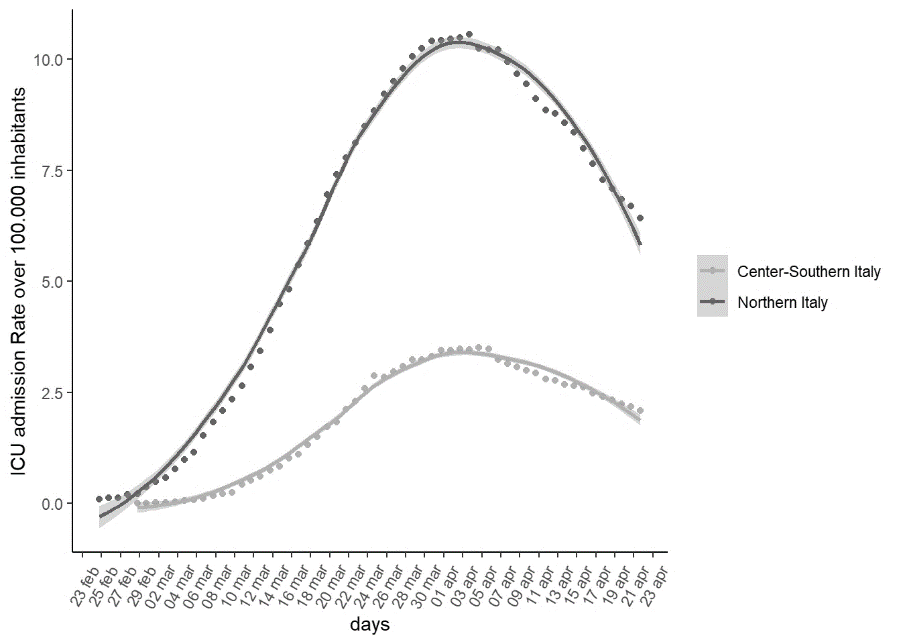  **Panel 1** Cumulative ICU admission rate over 100 000 inhabitants according to time in days and geographical area. A Local polynomial regressive smoothing spline has been also reported | |
| --- | --- |
| 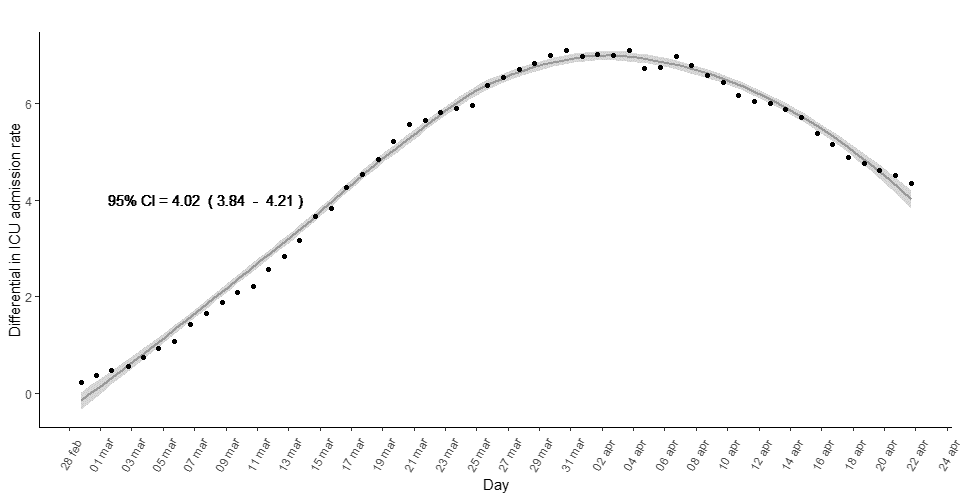  **Panel 2** Time series of the differential in ICU admission rate between Northern and Southern Italy | 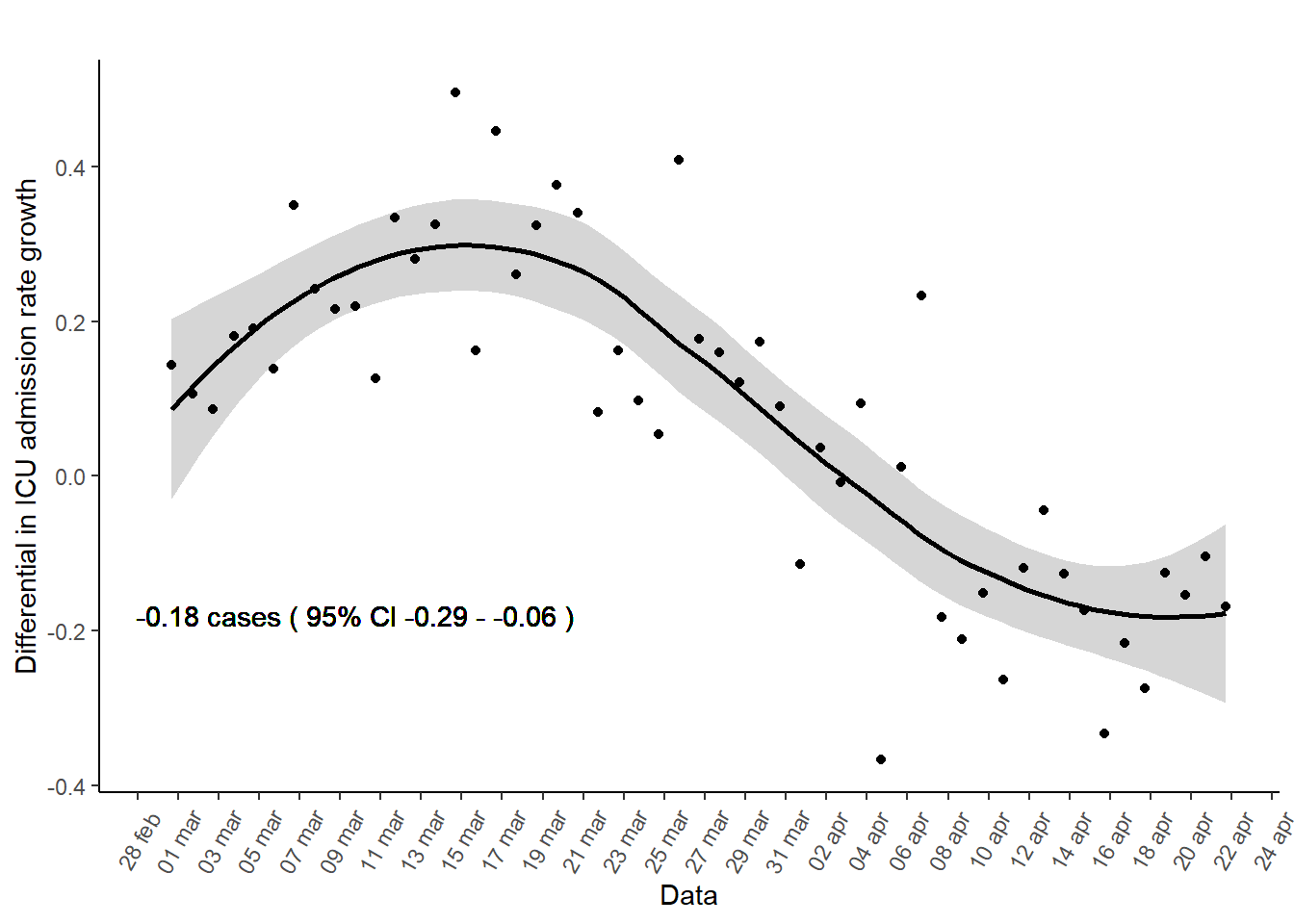  **Panel 3** Time series of the differential in ICU admission rate daily growth between Northern and Southern Italy. |

Table S3 Differential in hospitalization rates among Northern and Southern Italy.

| 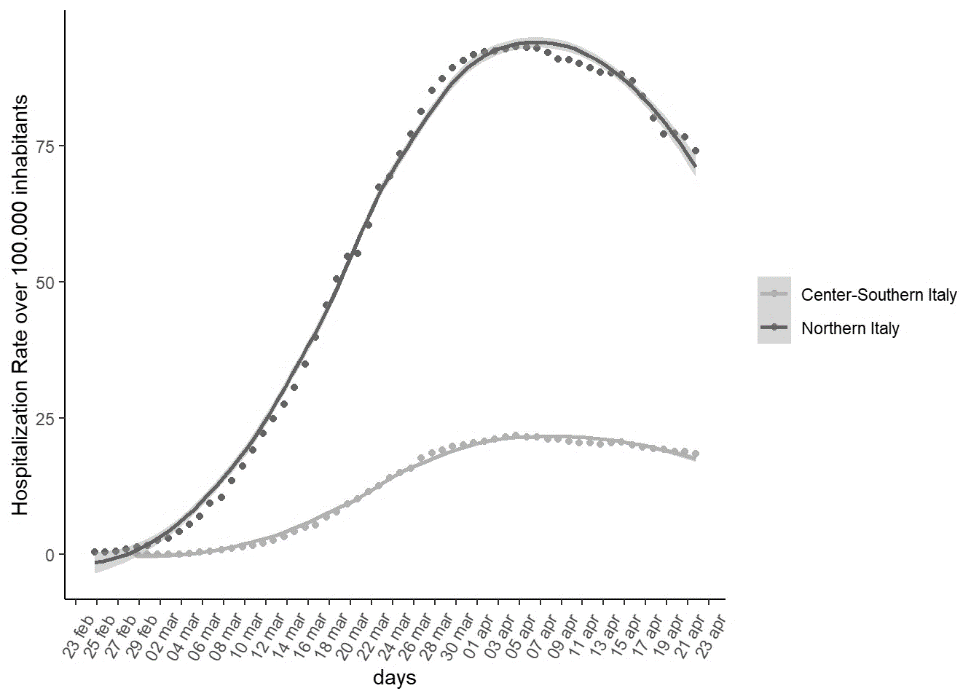  **Panel 1** Cumulative hospitalization rate over 100 000 inhabitants according to time in days and geographical area. A Local polynomial regressive smoothing spline has been also reported | |
| --- | --- |
| 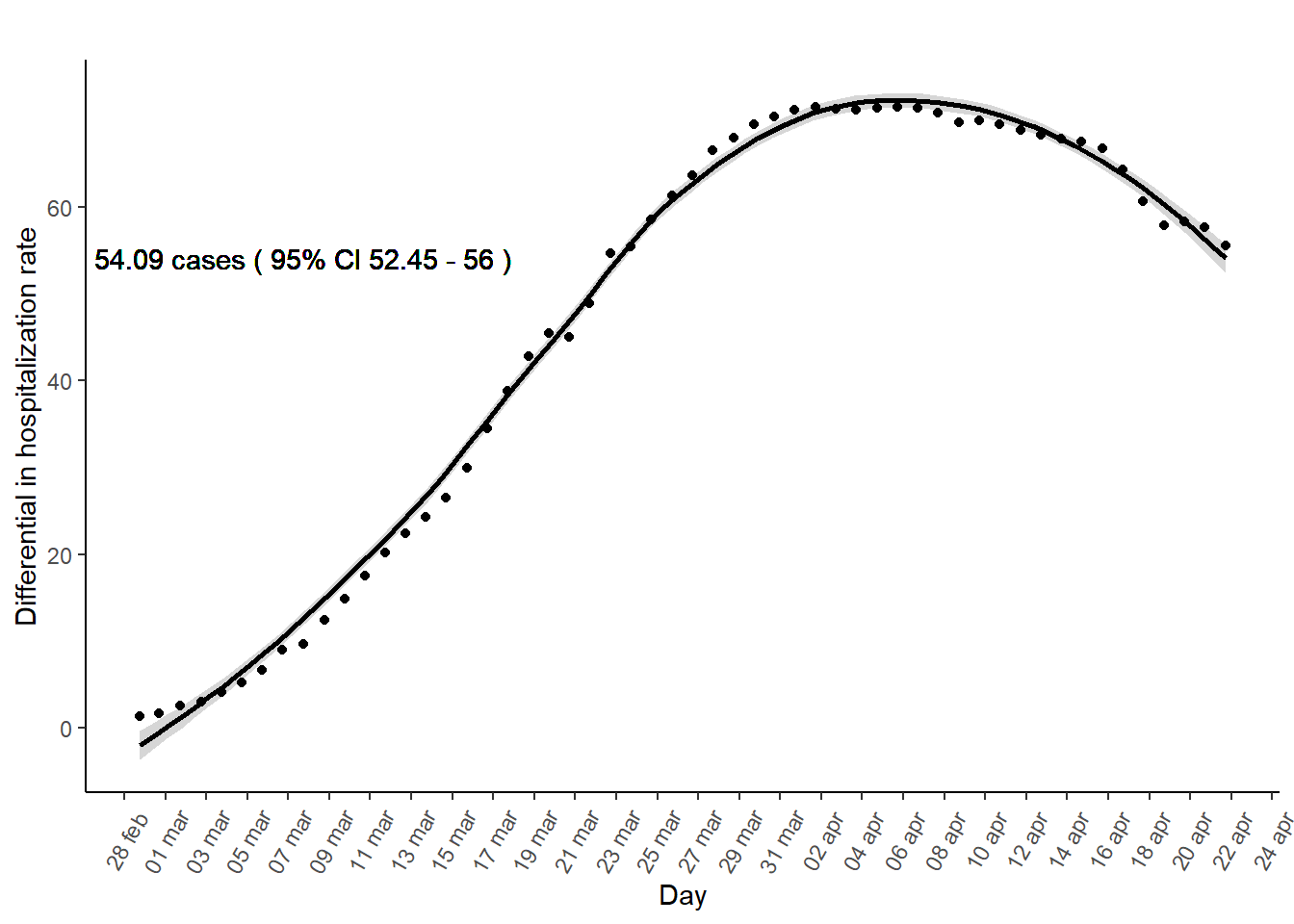  **Panel 2** Time series of the differential in hospitalization rate between Northern and Southern Italy | 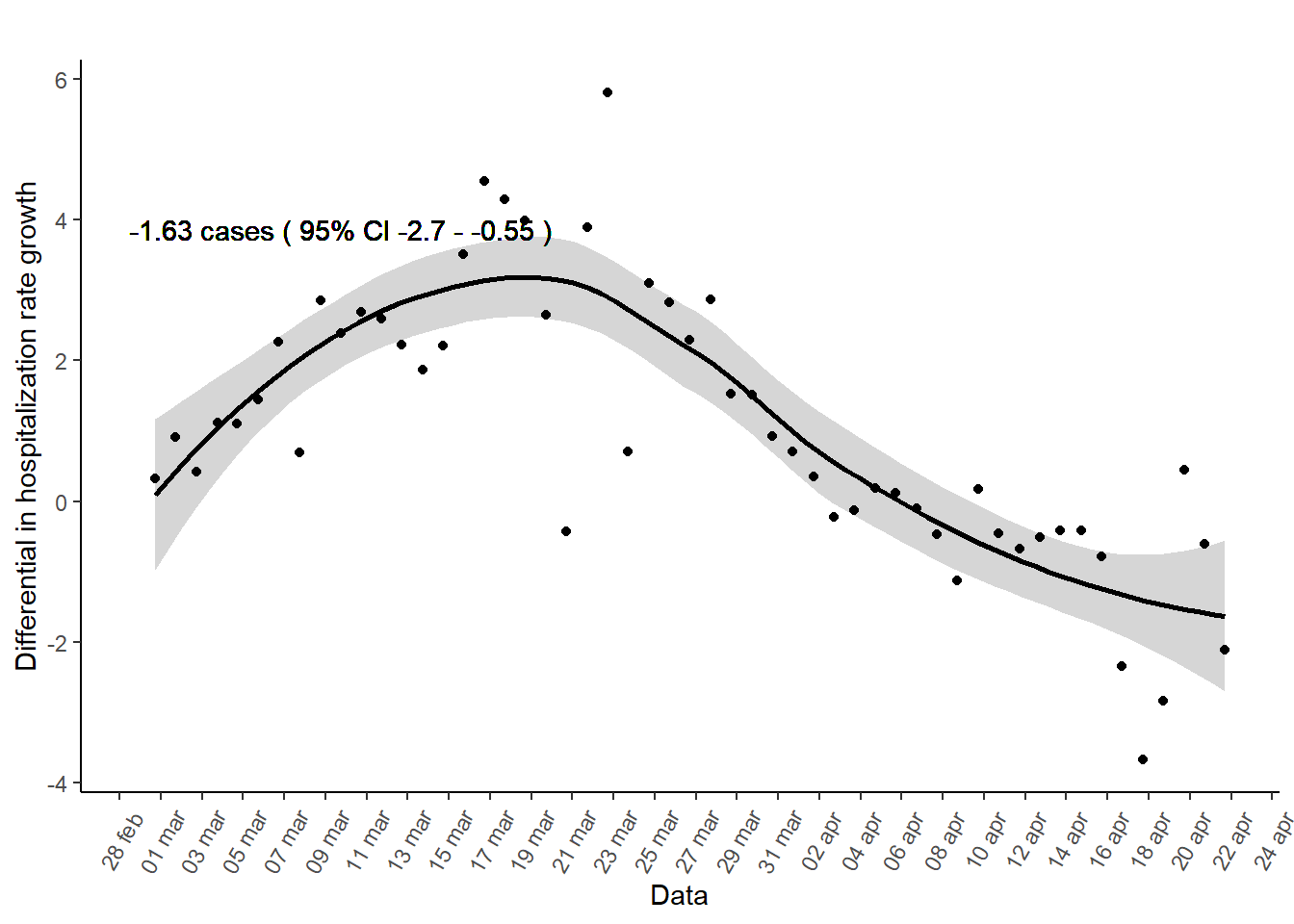  **Panel 3** Time series of the differential in hospitalization rate daily growth between Northern and Southern Italy. |


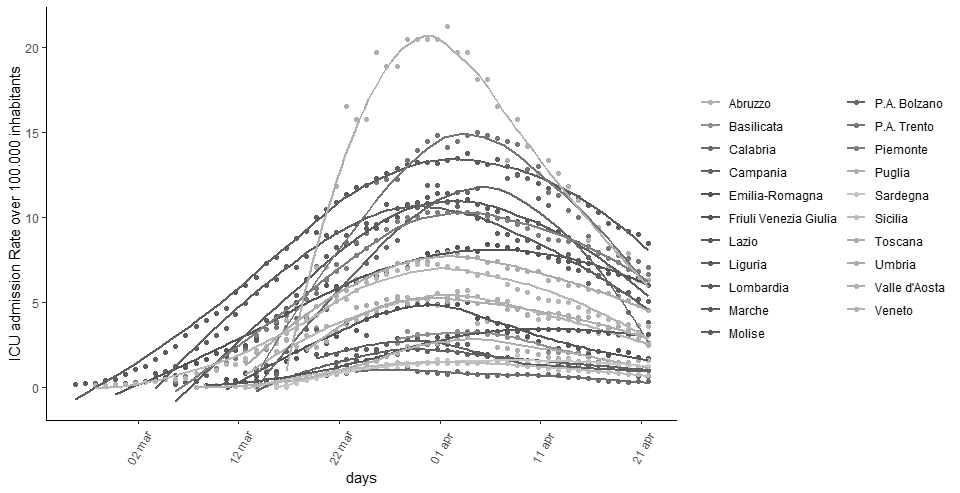


Figure S1 Observed ICU Admissions over 100 000 inhabitants


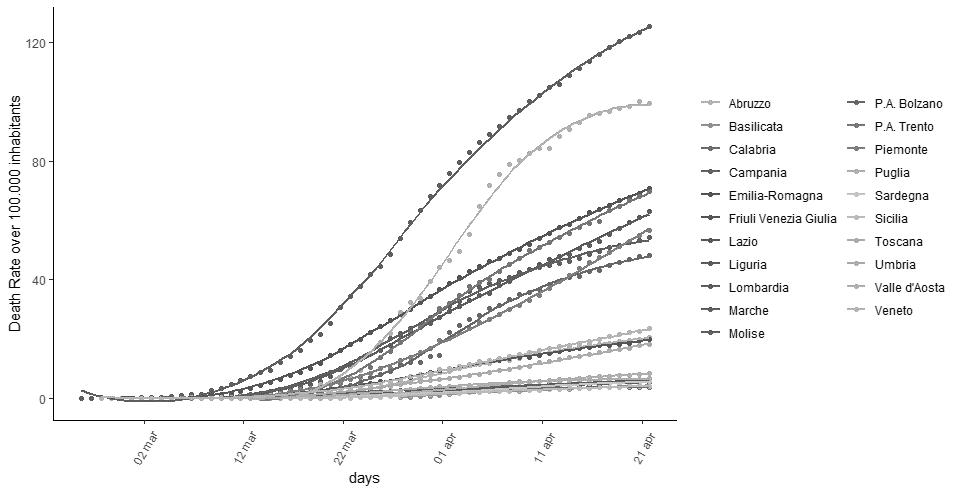


Figure S2 Observed mortality rate per 100 000 inhabitants


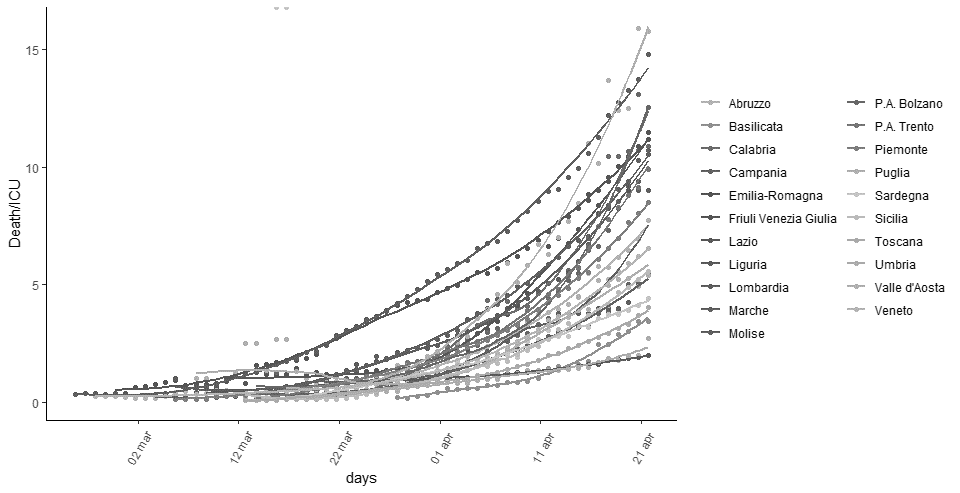


Figure S3 Observed Death ICU ratio


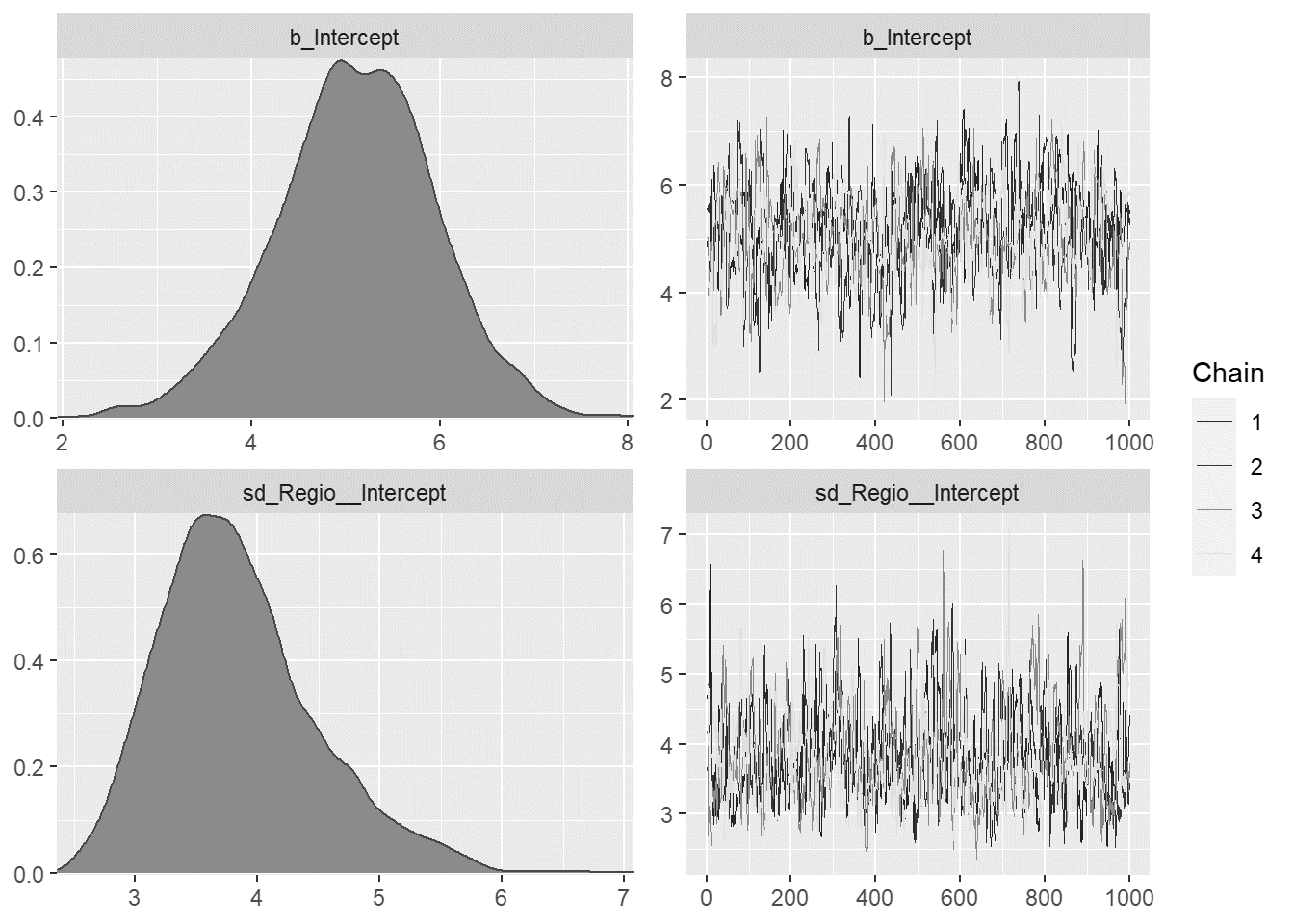


Figure S4 Posterior distribution and trace plots diagram ICU rate Model


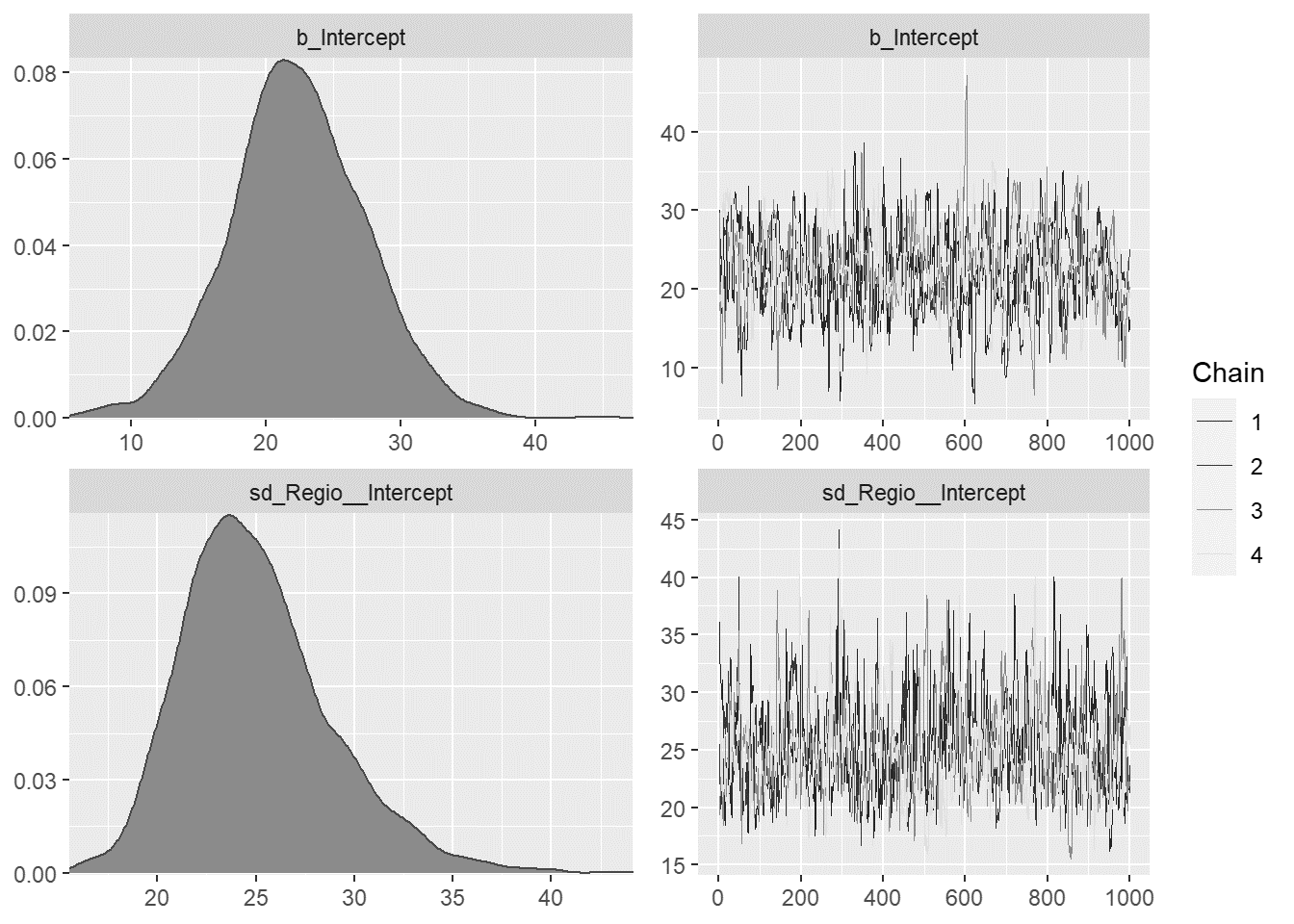


*Figure S5 Posterior distribution and trace plots diagram mortality rate Model*


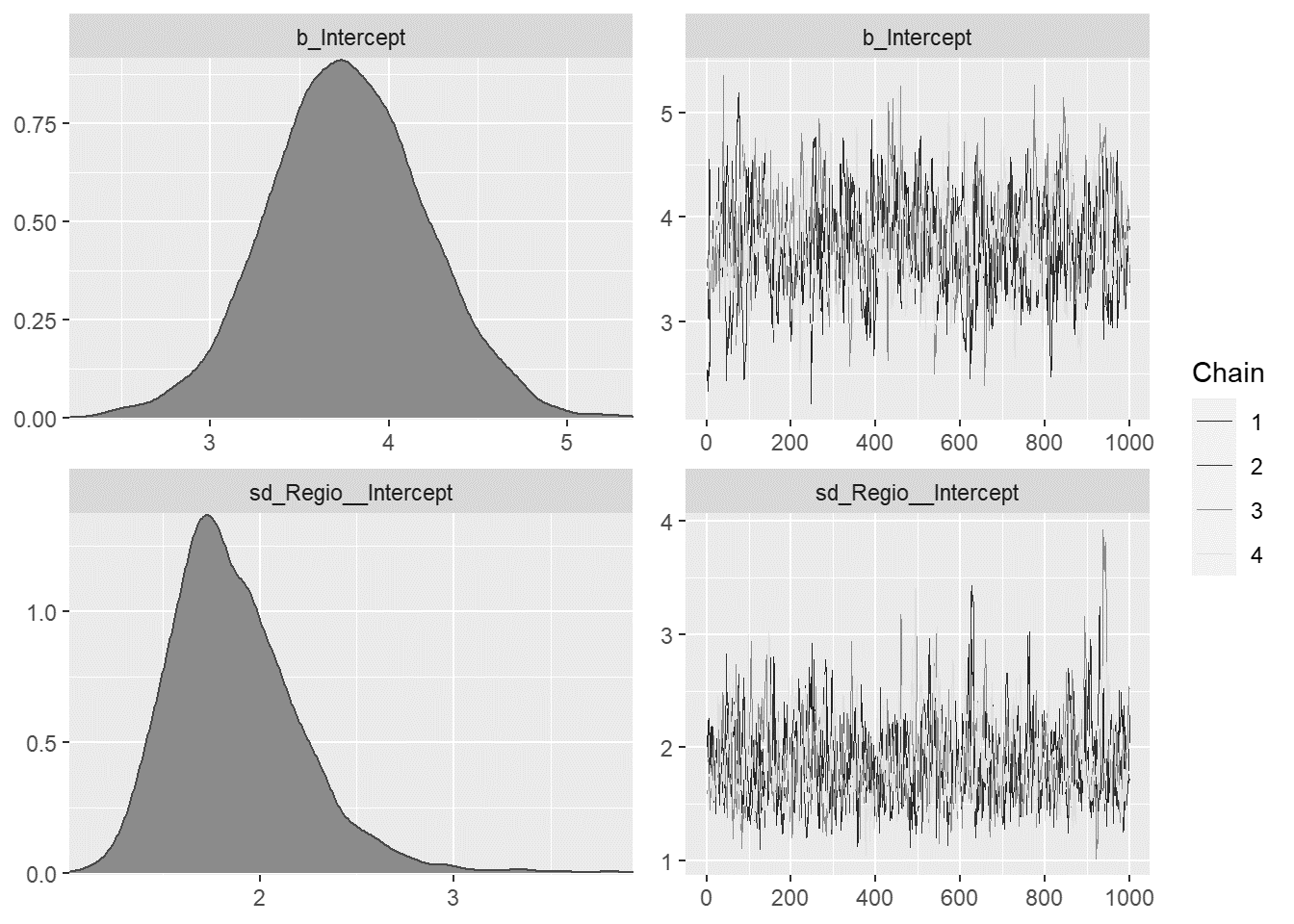


Figure S6 Posterior distribution and trace plots diagram Death over ICU Model
